# Bio-Guided Fractionation of Papaya Leaf Juice for Delineating the Components Responsible for the Selective Anti-proliferative Effects on Prostate Cancer Cells

**DOI:** 10.3389/fphar.2018.01319

**Published:** 2018-11-16

**Authors:** Saurabh Pandey, Carina Walpole, Paul N. Shaw, Peter J. Cabot, Amitha K. Hewavitharana, Jyotsna Batra

**Affiliations:** ^1^School of Pharmacy, The University of Queensland, Brisbane, QLD, Australia; ^2^School of Biomedical Sciences, Institute of Health and Biomedical Innovation, Queensland University of Technology, Brisbane, QLD, Australia; ^3^Australian Prostate Cancer Research Centre, Translational Research Institute, Queensland University of Technology, Brisbane, QLD, Australia

**Keywords:** anti-proliferative, bioassay guided fractionation, CyQUANT assay, papaya, prostate cancer

## Abstract

Alternative therapies against cancer cells with minimal or no effect on healthy tissues are highly sought after. Prostate cancer (PCa) is the second most frequently diagnosed malignancy in males. The *Carica papaya* L. leaf extract has been traditionally used by Australian aboriginal people for anticancer properties. In this study, medium polar fraction of papaya leaf extract that had shown anti-proliferative activity in PCa cell lines *in vitro*, in earlier studies, was further fractionated to 28 fractions by semi-preparative HPLC. Nine of these fractions were identified to possess selective anti-proliferative responses on PCa cells in comparison to non-cancerous cells of prostate gland origin. When these nine sub-fractions were mixed in various combinations, a combination containing six of the specific fractions (FC-3) showed the best potency. FC3 inhibited the growth of BPH-1, PC-3, and LNCaP cells in a concentration-dependent manner with an IC_50_ value <20 μg/mL, while (unlike paclitaxel, the positive control) minimal effect was observed on the proliferation of non-cancerous, WPMY-1 and RWPE-1cells. Furthermore, synergistic interaction of FC-3 with paclitaxel was observed with combination index values in the range of 0.89–0.98 and 0.85–1.10 on PC-3 and LNCaP cells, respectively. Untargeted qualitative analysis using UHPLC (Ultra High-Performance Liquid Chromatography)-QToF (Quadrupole Time of-Flight) mass spectrometry and screening against the METLIN database indicated presence of multiple known anticancer compounds in the FC-3 extract. These outcomes show that the potent and selective anti-proliferative effects are due to a range of bio-active compounds within the medium polar fraction of papaya leaf juice.

## Introduction

Plant derived drugs (vincristine, vinblastine, etoposide, taxol) are widely used to treat cancer. However, their dose associated side effects, and toxicity to non-tumor tissues negatively affect their utility. Therefore, alternative therapies against cancer cells with minimal or no effect on healthy tissues are highly desirable. A currently useful strategy used to evaluate herbal extracts is to test *in vitro* (Hoelder et al., 2012) for their selective anti-proliferative activities against cancer cells in comparison to normal cells. Various scientific studies have reported potent and selective growth inhibitory activities for plant extracts (Pandey et al., 2015). Effective compounds in plant extracts may act through various pathways to produce the desired biological responses. On the other hand, undesirable/inhibitory compounds in the same extract may produce toxic or antagonistic responses. Therefore, to reduce such antagonistic effects of plant extracts and to improve their potency, the fractionation and standardization of these preparations necessary (Yang et al., 2015).

Papaya (*Carica papaya* L.) leaves have a long history of nutritional and medicinal uses. In various countries, papaya leaves have been used as food (e.g., as salad in Vietnam and Indonesia). Papaya leaf extract has been reported to be traditionally consumed by Australian aboriginal people for its anti-cancer activity (Otsuki et al., 2010; Nguyen et al., 2013). A patent by Morimoto and Dang (2008) reported several case studies where consumption of aqueous papaya leaf extracts increased long-term survival of patients suffering from different cancers (stomach, pancreatic, lung, liver, and blood). Recently, Nguyen et al. (2016) reported the selective cytotoxic activities of lyophilised papaya leaf juice extract (LJP) on an oral squamous cell carcinoma (SCC25) in comparison to non-cancerous keratinocytes (HaCaT). Our previous study reported selective anti-proliferative and anti-metastatic attributes of LJP fraction(s) on prostate cancer (PCa) cells (Pandey et al., 2017). The lyophilised medium polarity fraction of papaya leaf juice (MP-LJP) yielded potent growth inhibitory activity (half-maximal growth inhibitory concentration, IC_50_ = 0.02–0.12 mg/mL) on benign prostate hyperplasia and number of PCa cell lines, with the exception of the normal (RWPE-1 and WPMY-1) cells. However, although the combination of compounds present in MP-LJP has selective anti-proliferative activity, there is also a possibility that some compounds present in MP-LJP might not contribute to this growth inhibitory activity. Our aim was to identify fractions of MP-LJP that are responsible for the selective anti-proliferative activity. In this study, we separated MP-LJP into a number of sub-fractions using semi-preparative HPLC, and tested each fraction and various combinations of fractions for their effects on cell viability.

With the increase in popularity of using herbal extracts as selective anti-cancer agents, it is an emerging strategy to incorporate herbal preparations or their bioactive compounds with chemotherapy drugs, thereby aiming for the reduction of dose-associated side effects and/or to enhance the efficacy of the drug (Yin et al., 2013). We used such a strategy by combining MP-LJP sub-fraction(s) with paclitaxel, a standard chemotherapy drug used to treat metastatic hormone-refractory PCa.

## Materials and Methods

### Plant Material and Preparation of MP-LJP

*Carica papaya* L., the only species within the genus *Carica* (Australian Government Office of the Gene Technology Regulator, 2008) was used for this study. Fresh papaya leaves of average maturity were collected from the “Tropical Fruit World,” Duranbah, NSW, Australia (28°17^′^15^′′^ S and 153°31^′^347^′′^ E). The MP-LJP was prepared by first rinsing leaves to remove any fine solids such as dust, followed by air drying to remove moisture from rinsing. It is important to remove water in order to prevent an aqueous (polar) extract instead of juice containing molecules of all polarities. The leaves (excluding veins) was processed using a pestle and mortar and the resulting juice was filtered through clean muslin cloth by a hand-pressing method. LJP (45 mL) was successively partitioned in a separating funnel using hexane and then ethyl acetate (EA), triplicate fractionations (15 mL × 3) of each to obtain a 45 mL fraction for each solvent. The MP-LJP was dried under vacuum at 40°C and stored at -80°C until further fractionation.

### Semi-Preparative HPLC Instrument

Medium polarity fraction of papaya leaf juice fraction was further fractionated on an Agilent 1200 series high-performance liquid chromatograph (Agilent Technologies, Inc., DE, United States), equipped with a vacuum degasser, binary pump, and diode array detector (set to a detection wavelength of 254 nm). The chromatographic separation was performed using a semi-preparative Eclipse XDB-C18, 7 μM, (21.2 × 250 mm) column (Agilent). The manually injected sample (20 mL) of MP-LJP (1 mg/mL) was chromatographed at a flow rate 10 mL/min, and at a column temperature of 25°C. Reservoir A contained water and reservoir B contained methanol. A 70 min binary linear gradient was employed: 70% B at 0 min, changing to 100% B in 5 min, followed by isocratic elution with 100% B for 55 min. The mobile phase composition was changed back to 30% A within 5 min, and the column was re-equilibrated with that mobile phase for 5 min. Sub-fractions were collected every 2.5 min using an automatic fraction collector. All 28 sub-fractions collected (25 mL each) were concentrated under vacuum at 40°C, and remaining solvent traces were removed using a temperature controlled Savant SpeedVac evaporator (Thermo Fisher Scientific, United States) in pre-weighed tubes. The dried sample tubes were weighed on a microbalance (Sartorius micro, Goettingen, Germany) to determine the weight of each sub-fraction. Dried sub-fractions (F1–F28) were stored at -80°C until further use.

### Cell Culture and Conditions

Prostate cell lines RWPE-1, WPMY-1, BPH-1, PC-3, and LNCaP were obtained from ATCC (American Type Culture Collection, VA, United States). RWPE-1 cells were cultured in keratinocyte-SFM media as recommended (Life Technologies, NY, United States). Other cells were cultured in phenol red-free RPMI1640 (1X) (Life Technologies, NY, United States) media supplemented with 5% v/v heat-inactivated fetal bovine serum and 1% v/v penicillin-streptomycin (Sigma, MO, United States) solution. All cell lines were maintained at 37°C in a humidified atmosphere containing 5% CO_2_ in CO_2_ incubator.

### Cell Proliferation Assay

The cells RWPE-1, WPMY-1, BPH-1, PC-3, and LNCaP cells were plated in 96-well black fluorescence micro-titre plates (PerkinElmer, VIC, Australia). For cell assays, the MP-LJP fraction and its sub-fractions were dissolved in DMSO to a concentration of 10 mg/mL. At 24 h after plating, triplicate wells were treated with the sterile filtered MP-LJP (0.002–0.02 mg/mL) and equivalent concentrations of MP-LJP sub-fractions. At the same time, cells were treated with positive control (paclitaxel, 100 nM), and negative control (0.3% v/v dimethyl sulfoxide, DMSO – Sigma, MO, United States). Cell proliferation assays using the CyQUANT NF cell proliferation kit (Molecular Probes, OR, United States) was performed to examine the anti-proliferative activity of the fractions. As per manufacturers protocol, CyQUANT dye was added to monitor growth of cells after 72 h intervals after treatment. Fluorescence was measured using a 96-well microplate reader FLUOstar Omega (BMG Labtech, Offenburg, Germany) with filters set at 480 nm excitation and 520 nm emission. The effects of MP-LJP sub-fractions on cell growth were calculated as fold changes in growth of extract treated cells vs. vehicle treated (0.3% DMSO) groups. Log concentration (inhibitor) vs. response (% growth change) curves was plotted, and non-linear regression analysis was performed to estimate half maximal growth inhibitory concentration (IC_50_) values. In addition, efficacy of treatments was reported as percent maximal growth inhibition (I_max_) post 72 h of exposure.

### Combination Concentration-Response Relationships of Paclitaxel and MP-LJP Sub-Fractions

Calculations of combination effects, based on the Chou-Talalay combination index (CI) method, were performed using CompuSyn software (ComboSyn Inc., NJ, United States) (Chou, 2010). The CI is a quantitative representation for drug interaction resulting additive effect (CI = 1), synergism (CI < 1), and antagonism (CI > 1). Following equation was used to calculate CI value:

$$\text{CI}=\sum_{j=1}^{n}\frac{{\left(f_{a}\right)}_{j}}{{\left(f_{u}\right)}_{j}}$$

Where n = number of drugs; f_a_ and f_u_ represents fraction affected and unaffected by dose, respectively.

### Chemical Profiling by LC-MS (QToF)

The Agilent 1290 UHPLC system (Agilent Technologies, CA, United States) was coupled to an Agilent 6520 high resolution accurate mass quadrupole time-of-flight (QToF) mass spectrometer equipped with a multimode source operating in both Electrospray Ionization (ESI) and Atmospheric Pressure Chemical Ionization (APCI) modes. The gradient method and mobile phase described above were used on a 2.1 × 50 mm Zorbax Eclipse plus (1.8 μm; C-18 RRHD) analytical column (Agilent) with C18 guard protection, for separations. Samples were dissolved in 70% v/v methanol and a 20 μL injection volume was used. A flow rate of 0.2 mL/min was used in all runs. Mass spectral acquisition was controlled using MassHunter software (B.02.01 SP3 – Agilent). The mass spectrometer was operated using a mass range of m/z 100–1700, at a scan rate of 3.0 cycles/s, and the following acquisition parameters: capillary voltage 2500 V, nebulizer pressure 30 psi, drying gas flow 5.0 L/min, gas temperature 300°C, fragmenting voltage 175 V, skimmer voltage 65 V.

Data processing and analysis for each extract (in triplicate) were performed using Agilent MassHunter Qualitative software (Version B.06.00) with Molecular Feature Extractor (MFE) algorithm. The following cut-off settings were employed: minimum peak filters of 500 counts, peak spacing tolerance of 0.0025 m/z plus 7.0 ppm, quality score >80%, minimum compound filters of 1000 counts. Mass Profiler Professional (MPP) software (Version 12.1 – Agilent) tool was used for binning, aligning, and creating a consensus for each feature. The MassHunter molecular formula generator (MFG) was used to generate a list of candidate molecular formulae (with ≥75% MFG score) using accurate mass values of metabolites. Further, putative compound identification of the features of interest was carried out using a Personal Compound Database Library (PCDL) (Agilent, United States) with the METLIN Personal Metabolite Database, and Naturally occurring Plant-based Anticancerous Compound-Activity-Target Database (NPACT) (Mangal et al., 2013).

### Data Analysis

Data were expressed as mean ± standard error mean (SEM, *n* = 3). Statistical differences compared between multiple groups of the MP-LJP fraction (and sub-fraction) treated groups and appropriate negative controls were analyzed by two-way analysis of variance (ANOVA) and followed by Bonferroni multiple comparison test. Log [inhibitor] vs. response curves were plotted and non-linear regression analysis with variable slope was performed to determine IC_50_ values. GraphPad Prism software (version 6.07) was used for all analyses.

## Results

### MP-LJP Sub-Fractions Have Selective Anti-proliferative Activities

**FIGURE 1:**
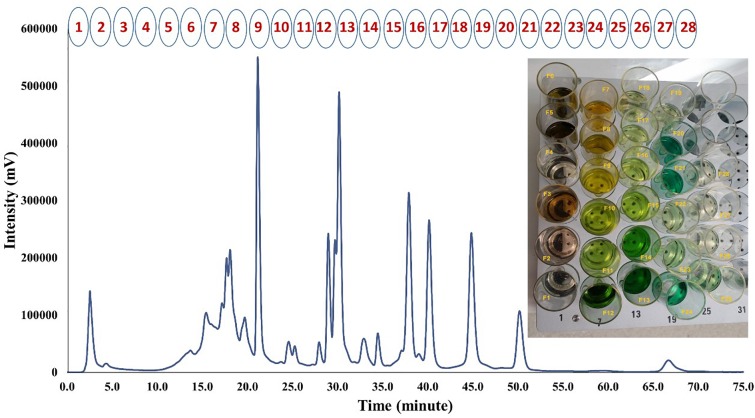
HPLC-UV chromatogram of MP-LJP at 254 nm and collected sub-fractions using automatic semi-preparative HPLC.

Medium polarity fraction of papaya leaf juice was sub-fractionated using reversed phase HPLC, with an optimized chromatographic gradient. Methanol/water mobile phase was used, and mobile-phase additives such as buffers were avoided. A total of 28 sub-fractions of MP-LJP were collected using semi-preparative chromatography, as illustrated in Figure 1. A cut-off time of 2.5 min (25 mL) was applied to collect sub-fractions (including all major UV peaks). There were few UV peaks that were divided when using this approach and were in sub-fraction combinations 1 and 2, 10 and 11, 12 and 13, and 18 and 19. However, it should be noted that many compounds do not necessarily produce a strong UV signal and therefore are not detected as peaks; they may, however, produce a signal in MS. This means that a method of collection of fractions based on UV peak position is less important when MS is used to identify compounds. In either case, some overlap is unavoidable with broad peaks. The percentage yield was calculated for each sub-fraction with respect to the dry weight of MP-LJP. Typically, the yield of MP-LJP was 3% w/w of lyophilised LJP (0.75 mg MP-LJP ≈ 25 mg LJP ≈ 1 g of fresh papaya leaf).

**FIGURE 2:**
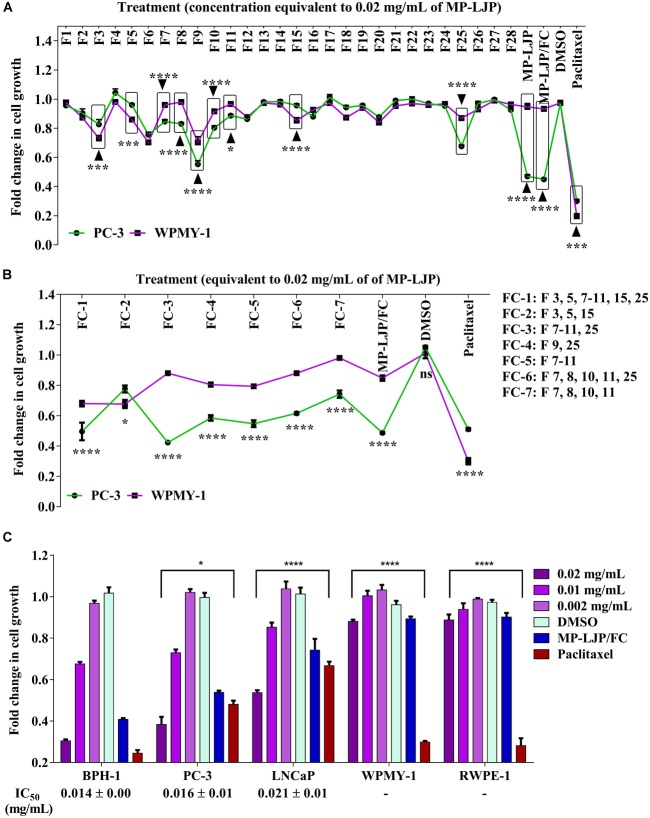
Anti-proliferative responses of MP-LJP sub-fractions after 72 h treatment on cancer cells and non**-**cancerous cells of prostate organ. CyQUANT NF proliferation assay was performed and the fold change in growth of fraction (FC)-treated cells with comparison vehicle (DMSO) treated cells was calculated. The data shown are mean ± SEM from three independent sets of three replicates. **(A)** Comparative responses of dried MP-LJP sub-fractions (F1–F28; 0.02 mg/mL), MP-LJP/FC and MP-LJP on PC-3 and WPMY-1 cells proliferation. **(B)** Anti-proliferative activity of sub-fraction in combination (F1: containing sub-fractions 3, 5, 7–11, 15, and 25; F2: sub-fractions 3, 5, and 15; F3: sub-fractions 7–11 and 25; F4: sub-fractions 9 and 25; F5: sub-fractions 7–11; F6: sub-fractions 7, 8, 10, 11, and 25; and F7: sub-fractions 7, 8, 10, and 11) on PC-3 cells with comparison to WPMY-1 cells. **(C)** Dose proliferation response of FC-3 after 72 h treatment of BPH-1, PC-3, LNCaP, PC-3, WPMY-1, and RWPE-1 cells. Non-linear regression analysis with variable slope was performed to determine IC_50_ values. Statistical analyses used two-way ANOVA followed by Bonferroni multiple comparison test with ^∗^*p* < 0.05, ^∗∗^*p* < 0.01, ^∗∗∗^*p* < 0.001, and ^∗∗∗∗^*p* < 0.0001, statistically different from vehicle treated cells.

The effects of fractions on the selective proliferative responses of bone metastasis derived PCa cell line, PC-3, in parallel to fibroblast cell line, WPMY-1 were examined (Figure 2). The anti-proliferative effects of intact MP-LJP were compared to the combination of all 28 sub-fractions (MP-LJP/FC); i.e., where the extract was re-constituted by combining all 28 fractions. No significant difference between activities of MP-LJP and MP-LJP/FC was observed, indicating no significant loss of bioactivity during the fractionation procedure (Figure 2A). Following 72 h of treatment, the selective growth inhibitory activities of MP-LJP sub-fractions (equivalent to 0.02 mg/mL of MP-LJP) on both cell lines were determined. Figure 2A displays the fold change in growth of PC-3 and WPMY-1 cells after treatment vs. vehicle (DMSO) treated cells. Among the 28 sub-fractions, sub-fractions 7, 8, 9, 10, 11, and 25 yielded significantly (*p* < 0.05) better anti-proliferative responses on PC-3 cells vs. WPMY-1 cells (with I_max_ values: 15% vs. 4%, 17% vs. 2%, 45% vs. 30%, 20% vs. 8%, 11% vs. 3%, and 32% vs. 13%, respectively). In contrast, sub-fractions 3, 5, and 15 displayed better growth inhibition of WPMY-1 cells vs. PC-3 cells (with I_max_ values: 27% vs. 17%, 14% vs. 4%, and 15% vs. 4%, respectively).

Several combinations of these 9 bio-active sub-fractions (3, 5, 7–11, 15, and 25)were also investigated to refine and identify the potent sub-fraction combination that gives the best growth inhibition of PC-3 cells relative to WPMY-1 (Figure 2B). Table 1 includes growth inhibitory efficacy (in terms of I_max_ values) of seven combinations of sub-fractions (FC), MP-LJP/FC and paclitaxel.

**Table 1:** Anti-proliferative responses of MP-LJP sub-fraction(s) in combination and paclitaxel on PC-3 and WPMY-1 cells.

Sub-fractions combination and paclitaxel (0.02 mg/mL of MP-LJP/FC)	% Maximal inhibition (I_max_)	
	PC-3	WPMY-1
**FC-1:** F3 + F5 + F7–11 + F15 + F25	50.38 ± 10.0	30.26 ± 1.8
**FC-2:** F3 + F5 + F15	22.36 ± 4.1	34.58 ± 2.7
**FC-3:** F7–11 + F25	57.68 ± 2.8	10.72 ± 0.8
**FC-4:** F9 + F25	41.49 ± 3.2	21.63 ± 1.4
**FC-5:** F7–11	45.24 ± 3.9	21.72 ± 1.0
**FC-6:** F7 + F8 + F10 + F11 + F25	38.46 ± 1.3	10.72 ± 0.8
**FC-7:** F7 + F8 + F10 + F11	25.79 ± 4.2	8 ± 0.8
**MP-LJP/FC:** F1–28	51.36 ± 1	12.06 ± 1.8
**Paclitaxel**	48.94 ± 1.5	67.24 ± 2.1

Among sub-fraction combinations (FC-1 to FC-7), FC-2 was found to be the least selective, while FC-3 displayed the best growth inhibitory effect, and the best selective growth inhibitory effect, in comparison to all combinations (and paclitaxel), against PC-3 cells vs. WPMY-1 cells. MP-LJP/FC (combination of all fractions of MP-PLJ), and FC-3 yielded similar growth inhibitory effects over PC-3 cells (I_max_ values 58% vs. 52%) and WPMY-1 cells (11% vs. 12%). This suggests that the bulk of the selective anti-proliferative properties contained in MP-LJP is localized in FC-3. The anti-proliferative effect (62% vs. 49% against PC-3 cells) and selective anti-proliferative effect (12% vs. 70% against WPMY-1 cells) of FC-3 were better than those of paclitaxel.

To calculate the anti-proliferative potency (IC_50_) of FC-3, a concentration proliferation response post 72 h treatment over the range 0.002–0.02 mg/mL (equivalent to dry weight of MP-LJP) was performed on BPH-1, PC-3, and LNCaP cells (Figure 2C). In addition, to confirm selective responses of FC-3, concentration proliferation response studies against non-cancerous cells were performed. FC-3 significantly inhibited the growth of BPH-1, PC-3, and LNCaP cells with an IC_50_ value 0.015, 0.016 and 0.021 mg/mL, respectively, and did not yield significant anti-proliferative effect on non-cancerous cells. In contrast, results from proliferative responses indicated significant growth inhibition of non-cancerous cells by paclitaxel. All three Figures 2A–C demonstrate the superior selective anti-proliferative effect of MP-LJP and FC-3 when compared to paclitaxel.

### FC-3 in Combination With Paclitaxel Yielded Synergistic Anti-proliferative Activity

**FIGURE 3:**
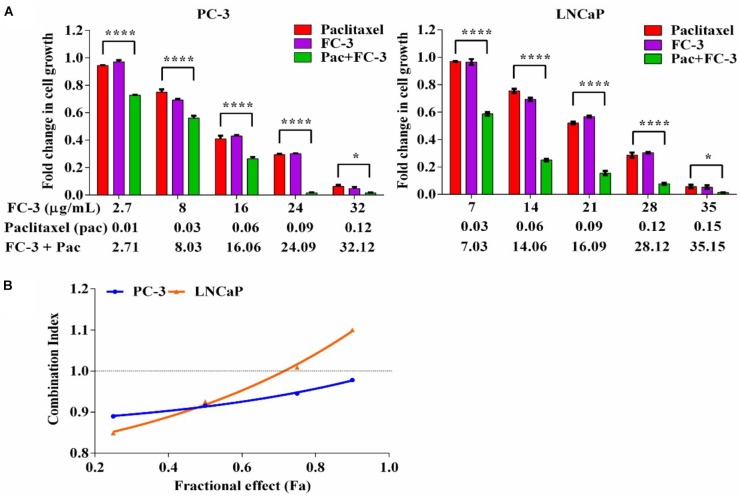
Combination effect of FC-3 and paclitaxel in prostate cancer cells (PC-3 and LNCaP). **(A)** Concentration-proliferation response graph, cells were treated with FC-3, paclitaxel, and the combination of both agents (molar ratio of the combination was IC_50_ FC-3: IC_50_ paclitaxel) for 72 h. Statistical analyses were done using two-way ANOVA followed by Bonferroni multiple comparison test with ^∗^*p* < 0.05, ^∗∗^*p* < 0.01, ^∗∗∗^*p* < 0.001, and ^∗∗∗∗^*p* < 0.0001, statistically different from paclitaxel treated cells (*n* = 3, mean ± SEM). **(B)** Combination index (CI) across the fraction affected (f_a_). The CI values were assessed using CalcuSyn software to determine the interaction (where CI > 1, =1, and <1 suggest antagonism, synergism, and additivity, respectively).

Following 72 h exposure, the growth inhibitory properties of FC-3 when in combination with paclitaxel on PC-3 and LNCaP cell lines were analyzed. A constant ratio design (using an IC_50_ value ratio of FC-3/paclitaxel, i.e., 16/0.06 and 21/0.09 on PC-3 and LNCaP cells, respectively) was used to systematically examine combination concentration-anti-proliferative response relationships between both agents. Combination data is presented in terms of FC-3 with paclitaxel (μg/mL) concentrations. As shown in Figure 3A, FC-3 in combination with paclitaxel significantly (*p* < 0.05) potentiated the growth inhibitory activity of paclitaxel. The proliferation of PC-3 cells decreased significantly from 94 to 6%, 97 to 5%, and 73 to 1% with increasing concentrations of paclitaxel (0.01–0.12 μg/mL), FC-3 (2.7–32 μg/mL) and combinations, respectively. Following treatment with paclitaxel (0.03–0.15 μg/mL), FC-3 (7–35 μg/mL) and in combination, growth inhibitory activities were observed on LNCaP cells with 97 to 6%, 97 to 5%, and 59 to 1%, respectively. The data from the proliferation study were analyzed using median-effect equation, and resulting plot of combination index (CI) vs. fractional affect (IC_25_, IC_50_, IC_75_, and IC_90_) was used to determine the type of interaction between FC-3 and paclitaxel (Figure 3B). With PC-3 cells, the combinations of FC-3 and paclitaxel displayed a modest synergistic interaction (CI: 0.89–0.98) at all concentration combinations tested. A similar synergistic interaction was observed on LNCaP cells (CI: 0.85 and 0.93 at 25 and 50% effect level, respectively). While at higher concentrations nearly additive effects (CI: ≤1.10) were induced by the combination in LNCaP cells.

### Tentative Identification of Bioactive Compounds in FC-3 Using LC-MS (QToF)

Untargeted data acquisition of samples in triplicate was performed using LC-MS (QToF) in positive and negative ion modes (Supplementary Figure S1). The untargeted data mining algorithm, MFE (molecular feature extraction), was used to extract all relevant spectral and chromatographic information (by finding the co-variant ions, specified adducts, and removing the background ions), as “features” for unknown compounds/metabolites. MPP (Mass Profiler Professional) software was used to generate normalized features list. A total of 922 and 1470 features were obtained for MP-LJP/FC in the positive and negative ionization modes, respectively. For FC-3, 347 and 558 features were obtained in positive and negative ionization modes, respectively. The MFG (molecular formula generation) algorithm was used to predict empirical molecular formulae for the normalized accurate mass values (by considering monoisotopic mass, isotope abundances, spacing between isotope peak information, and user specified elements) with corresponding MFG scores (i.e., the relative probability leading to correct formula). Subsequent comparison in conjunction with MFG conditionality (≥75% MFG score) resulted in lists of 108 (positive ion mode) and 172 (negative ion mode) features for FC-3 (Supplementary Tables S1, S2). The resultant list was exported and searched against the METLIN Personal Metabolite Database (accessed on July 2016), yielding 822 and 673 compounds within mass tolerance window ≤5 ppm. A large number of hits were observed for some single mass values; e.g., more than 50 different compounds each were recovered as matches for mass values 389.2695, 401.3415, and 435.3477 in positive ion mode, and similarly for the mass values 233.155, 311.223, 315.198 in negative ion mode. Further, the comparison of compound list against NPACT database (which contains 1,574 entries of plant-derived natural compounds with known anticancer activity) resulted in 21 matched anticancer compounds (Table 2).

**Table 2:** Tentative identification of compounds in FC-3.

Experimental mass	Retention time (min.)	Empirical formula	Error (ppm)^@^	Number of hits^#^	Putative anticancer compounds^$^
238.2289	7.764	C_16_H_30_O_2_	1	34	Hexadecenoic acid
278.225	4.542	C_18_H_30_O_2_	0	78	Alpha and gamma linolenic acid
284.2356	7.043	C_17_H_32_O_3_	1	10	Heptadec-16-yne-1,2,4-triol
284.2718	8.495	C_18_H_36_O_2_	0	19	Stearic acid
272.178	3.371	C_18_H_24_O_2_	0	20	(2S,4aS,10aR)-1,1,4a,7-tetramethyl-2,3,4,10a-tetrahydrophenanthrene-2,6-diol; Octadeca-9,11,13-triynoic acid
592.2689	8.992	C_35_H_36_N_4_O_5_	0	1	Pheophorbide A
250.157	2.598	C_15_H_22_O_3_	0	30	Viscic acid
234.162	2.71	C_15_H_22_O_2_	0	58	(1R,4E,9S)-4,11,11-trimethyl-8-methylidenebicyclo[7.2.0]undec-4-en-3-one
384.3394	25.203	C_27_H_44_O	0	43	Vitamin D
416.3653	11.891	C_28_H_48_O_2_	0	16	Gamma-Tocopherol
316.205	5.339	C_20_H_28_O_3_	2	57	8,14-epoxide; Caracasine acid; Multidione; Rabdoumbrosanin; (+)-7-Oxo-13-epi-pimara-14,15-dien-18-oic acid; (+)-7-Oxo-13-epi-pimara-8,15-dien-18-oic acid; Taiwaniaquinone G
440.3655	8.81	C_30_H_48_O_2_	0	16	Friedelan-1,3-dione
324.194	3.001	C_18_H_28_O_5_	0	5	3-alpha-acetoxydiversifolol

*^@^Error (ppm): the difference between experimental mass and theoretical mass of compound/theoretical mass of compound; ^#^Hits obtained from METLIN database; ^$^Putative compounds from NPACT database*.

## Discussion

It has become increasingly popular to utilize plant-based preparations and their associated compounds against various ailments, and cancer is no exception. The potential medicinal importance of *C. papaya* against different cancers is evident from a variety of *in vitro* studies (Nguyen et al., 2013). A number of studies have reported selective cytotoxic responses of papaya extracts against breast (MCF-7 and MDA-MB-231) and skin (SCC 25) cancer cells, with minimal effects on non-tumor cells of breast (MCF-12F) and skin (HaCaT) (Garcia-Solis et al., 2009; Nguyen et al., 2015, 2016). Following a previous report, where selective anti-proliferative responses of medium polarity fraction (MP-LJP) were observed on a range of PCa cells (Pandey et al., 2017), the present study focused on additional fractionation of MP-LJP fraction to seek to identify specific compounds that may induce selective anti-proliferative activities on benign and PCa cells, by comparison with non-cancerous cells of prostate origin.

In order to determine the respective sub-fractions of MP-LJP responsible for selective growth inhibitory activity, range of cells representing benign, cancer and non-cancerous cells of prostate origin were selected and analyzed. A previous study has reported IC_50_ values for MP-LJP on benign and PCa cells in the range of 0.02–0.07 mg/mL (Pandey et al., 2017). Based on these results, 0.02 mg/mL of MP-LJP and equivalent amounts (i.e., equivalent to 0.02 mg/mL of MP-LJP) of sub-fraction(s) were chosen for initial experiments on PC-3 and WPMY-1 cells. It is important to consider the potential cytotoxic and growth inhibitory activity of organic solvents used for the fractionation and solubilisation of plant extracts. Control experiments were carried out using an amount of methanol equivalent to the highest concentration in fractions dissolved in DMSO, and using an amount of DMSO equivalent to the highest concentration in fractions. No significant effects were observed on the growth of cells after 72 h vehicle treatment, i.e., DMSO in media and DMSO containing methanol in media (data not shown).

It is possible that the fractionation process may have affected the stability of the plant’s bioactive compounds (Houghton, 2000). Various studies have reported losses of bioactivity following bioassay-guided fractionation (Jackson et al., 2000; Liu et al., 2005). However, when we compared the anti-proliferative activities of MP-LJP and combination of all sub-fractions (MP-LJP/FC) on PC-3 and WPMY-1 cells, no major differences in anti-proliferative responses were observed (Figure 2A). This result suggests that the reversed phase preparative chromatography fractionation process used in this study did not lead to any loss of MP-LJP bioactive compounds. The MP-LJP sub-fraction(s) associated with selective anti-proliferative property were identified and, as illustrated in Figure 2A, the MP-LJP sub-fraction(s) F3, 5, 7–11, 15, and 25 displayed selective growth inhibitory properties.

The observation that many sub-fractions with growth inhibitory activity did not elute close to each other suggests that they are likely to have differing polarities and thereby differing chemical compositions. These results indicate the involvement of a range of active components in the inhibition of PCa cell growth. Furthermore, the finding that no single sub-fraction of MP-LJP displayed similar effects to those of the parent fraction indicates that further separation of these sub-fractions would not be helpful in the delineation and enhancement of anti-proliferative activity. Therefore, combinations of the sub-fraction(s) were tested to identify those bioactive fractions of MP-LJP that were responsible for selective growth inhibition. Only the sub-fraction(s) that displayed selective growth inhibitory activity, i.e., F3, 5, 7–11, 15, and 25 were chosen for the combination study. Several combinations, including all sub-fractions, based on their individual selective growth inhibitory activities were tested as shown in Table 1. The sub-fraction combination of FC-3 containing sub-fractions F7–11 and F25 resulted in a significant selective anti-proliferative response; this effect was found to be similar to that of MP-LJP/FC (Figure 2B) on PCa cell proliferation. This demonstrates that the bulk of the potency of MP-LJP is contained in the fraction combination FC-3. The NCI (United States National Cancer Institute) recognizes as an effective preparation, any plant product that display anticancer activity with an IC_50_ value ≤0.02 mg/mL (Sudarshana, 2012). Results from concentration effect analysis of FC-3 indicated its promising growth inhibitory property (with IC_50_ values ≤0.02) on BPH and PCa cells. With comparison to parent fraction, FC-3 induced similar responses on BPH-1 (0.014 mg/mL vs. 0.011 mg/mL) and PC-3 (0.016 mg/mL vs. 0.02 mg/mL) cells, and a significantly (*p* < 0.001) high response on LNCaP cells (0.021 mg/mL vs. 0.07 mg/mL). Moreover, no effect was observed on non-cancerous cells of prostate gland origin (Figure 2C). These responses may be due to synergistic, additive and/or antagonistic interactions among compounds (Rasoanaivo et al., 2011) present in FC-3 combination fraction of MP-LJP on diseased and normal cells of prostate.

Plant preparations are recognized as a useful complementary medicine for the treatment of cancer. Many scientific studies have attempted to enhance the therapeutic efficacies (and reduce the side effects) of chemotherapeutic agents by incorporating promising plant preparation (Yin et al., 2013). We have demonstrated here, for the first time, the synergistic interaction of LJP extract with paclitaxel has potent anti-proliferative activity on PCa cells. A concentration-dependent increase in anti-proliferative effect was achieved on PC-3 and LNCaP cells when sub-fraction combination FC-3 of MP-LJP was combined with paclitaxel (Figure 3). This synergistic response shows the potential of using a lower paclitaxel concentration to achieve the same level of effectiveness as shown by a high paclitaxel concentration used alone. However, to further understand the wide spectrum adjuvant role of FC-3 in the reduction of side effects (i.e., toxicity to other cells and the development of resistance) and the increase in the therapeutic efficacy of chemotherapy, a combination study using other chemotherapeutic drugs over different cancer cells is required.

The chemical diversity of plant extracts with bioactivities of interest provides many opportunities for drug development. Mass profiles representing different compounds in MP-LJP/FC and FC-3 were acquired using UHPLC-QToF. The accurate mass measurement along with prediction of molecular formula of compounds is an efficient way of untargeted metabolomic analysis of plant preparations (Sana et al., 2008; Wu et al., 2016). Data analysis (using software MFE, MPP, and MFG) of FC-3 was performed and putative lists of compounds present in FC-3 were generated (Supplementary Tables S1, S2). Each individual feature corresponds to a large number of potential compounds. The most likely molecule was identified based on the scores given by the MFE followed by the MFG software; thus a number of other compounds were eliminated at this stage (Supplementary Figure S2). The detected compounds using the METLIN database were matched against the NPACT database for putative identification of plant-based known anticancer compounds. The search revealed the presence of anticancer compounds from classes of fatty acids, aliphatic alcohol, terpenoids, vitamins, and a chlorophyll breakdown product (pheophorbide A) (Mangal et al., 2013).

In a previous report, where the acidic ethanol extract of papaya leaf induced an increased cytotoxic activity on skin cancer cells when compared to the normal cells of skin, the extract contained flavonoids and phenolic acids (Nguyen et al., 2015). Although there is a possibility that the same and/or similar compounds may be present in medium polarity LJP extract (due to the solubility of medium polar compounds in ethanol), there are significant differences in the tentative lists of compounds observed in this paper and those reported above. This may be due to a potential decreased abundance of polar compounds in MP-LJP (and FC-3) when compared to those in an ethanolic papaya leaf extract. The FC-3 fraction in this study showed a greater efficacy and selectivity (IC_50_ = 0.02–0.07 mg/mL) when compared to the acidic ethanol extract of papaya leaf (IC_50_ = 0.77 mg/mL) (Nguyen et al., 2015) – albeit on different cell lines, thereby potentially indicating differences in the compositions of FC-3 and the acidic ethanol extract. Compounds such as hexadecenoic acid, stearic acid, linolenic acid, terpenoids, pheophorbide A and vitamins have been previously documented in papaya leaf extracts (Melariri et al., 2011; Mohamed, 2013; Maisarah et al., 2014; Nguyen et al., 2016). The few studies that have examined the biological activities of medium polarity papaya leaf extracts reported the presence of aliphatic fatty acid (linoleic acid and linolenic acids), and an alkaloid (carpaine) (Melariri et al., 2011; Mohamed, 2013). Similar to those studies, here we observed the possible presence of linolenic acid (m/z 279.2321) and carpaine (m/z 479.3838) in FC-3. The anticancer activities of linolenic acid and its role in the modulation of the cytotoxic responses of various chemotherapeutic drugs are well known (Menendez et al., 2001). A review article that focused on studies associated with the cytotoxic activity of fatty acids (especially omega-3 fatty acids), *via* apoptosis of cancer cells, has suggested their potential cytotoxic role (by targeting multiple molecular signals involved in tumor cell death) in multi-targeted cancer therapy (D’Eliseo and Velotti, 2016). A study by Nikolakopoulou et al. (2013) has reported a selective growth inhibitory response of omega-3 polyunsaturated fatty acids in neoplastic oral keratinocytes by differentially activating ERK1/2 (Nikolakopoulou et al., 2013). Apart from fatty acids, a piperidine alkaloid carpaine (0.93 g/kg of papaya leaf) (Wang et al., 2015) a potential proteasome inhibitor was suggested to be a potential anticancer agent (Fahy et al., 2004). A recent study has also confirmed the presence of pheophorbide A in LJP that displayed cytotoxic activity on skin cancer cells (Nguyen et al., 2016). Pheophorbide A has been reported to be a substrate of ABCG2 (breast cancer resistance protein). Human ABCG2 protein is widely expressed in normal tissues, and suggested to provide first line of defense for normal cells from environmental stress (Mo and Zhang, 2012).

All the putative anticancer compounds in Table 2 are physicochemically medium-polar to non-polar in nature, thus an indicator for the presence of these compounds in FC-3. It should be noted that the abundance of compounds assigned on the basis of peak areas, and peak area is not necessarily reflective of the abundance – some of the most abundant compounds may have very low ionization efficiency in MS source producing small peaks. Future studies by comparison with reference compounds and using complementary analytical techniques (such as NMR to narrow down the list of hits for each molar mass based on structural information) are required to confirm the identities of FC-3 associated compounds that are responsible for the selective anti-proliferative responses on PCa cells. It should be noted that the elimination of features/compounds at each stage of data mining, as described above, may eliminate some potent (but with low abundance or with low ionization efficiency) anti-cancer compounds from their identification as components of FC-3. Furthermore, as only a fraction of compounds that progressed beyond the data mining process have been identified using the databases, there is a good chance that the potent anti-cancer compounds in FC-3 are, as yet, not identified because they are not previously recorded and therefore not listed in databases. These represent some of the limitations in identification of bio-active compounds using currently available technology. Another possible complication is the possibility of metabolic transformation of bioactive compounds by cellular enzymes (Aragonès et al., 2017). This will add a limitation to the identification of bio-active compounds through databases.

## Conclusion

Bioassay-guided fractionations of medium polarity LJP resulted in several sub-fractions of MP-LJP that showed selective anti-proliferative responses against PCa cells (including benign hyperplasia) in comparison to non-cancerous cells of prostate origin. Studying several combinations of these sub-fractions resulted in a highly refined FC-3 extract that accounted for bulk of the potent and selective anti-proliferative responses of MP-LJP. The anti-proliferative, and selective anti-proliferative responses of FC-3 were superior to those of Paclitaxel. Furthermore, FC-3 extract in combination with paclitaxel induced synergistic growth inhibitory effects on PCa cells. Untargeted qualitative analysis using UHPLC-QToF indicated the presence of multiple known anti-cancer compounds in FC-3 extract. These results show the potential of FC-3 for cancer therapy, after conducting the appropriate and necessary *in vivo* efficacy studies on prostate tumor animal models.

## Author Contributions

AH, JB, SP, CW, PS, and PC conceived and designed the experiments. SP performed the experiments, wrote the manuscript, and discussed the results. AH, PS, and JB contributed to reagents, materials, and analysis tools. JB, AH, and PS critically reviewed the manuscript.

## Conflict of Interest Statement

The authors declare that the research was conducted in the absence of any commercial or financial relationships that could be construed as a potential conflict of interest.
