# Supplementary material for: Bio-Guided Fractionation of Papaya Leaf Juice for Delineating the Components Responsible for the Selective Anti-proliferative Effects on Prostate Cancer Cells
Source: Front Pharmacol. 2018 Nov 16;9:1319. doi: 10.3389/fphar.2018.01319 (PMC6250729; doi:10.3389/fphar.2018.01319)
Supplement: Supplementary file 1 [file Table_1.DOCX]

***Supplementary Material***

**Bio-Guided Fractionation of Papaya Leaf Juice for Delineating the Components Responsible for the Selective Anti-Proliferative Effects on Prostate Cancer Cells**

Saurabh Pandey^1,2,3^, Carina Walpole^2,3,^ Paul N. Shaw^1^, Peter J. Cabot^1^_,_

Amitha K. Hewavitharana^1^, Jyotsna Batra^2,3*^

*Correspondence:

Dr Jyotsna Batra ( [jyotsna.batra@qut.edu.au](mailto:jyotsna.batra@qut.edu.au))

Dr Amitha Hewavitharana (ahewavitharana@pharmacy.uq.edu.au)


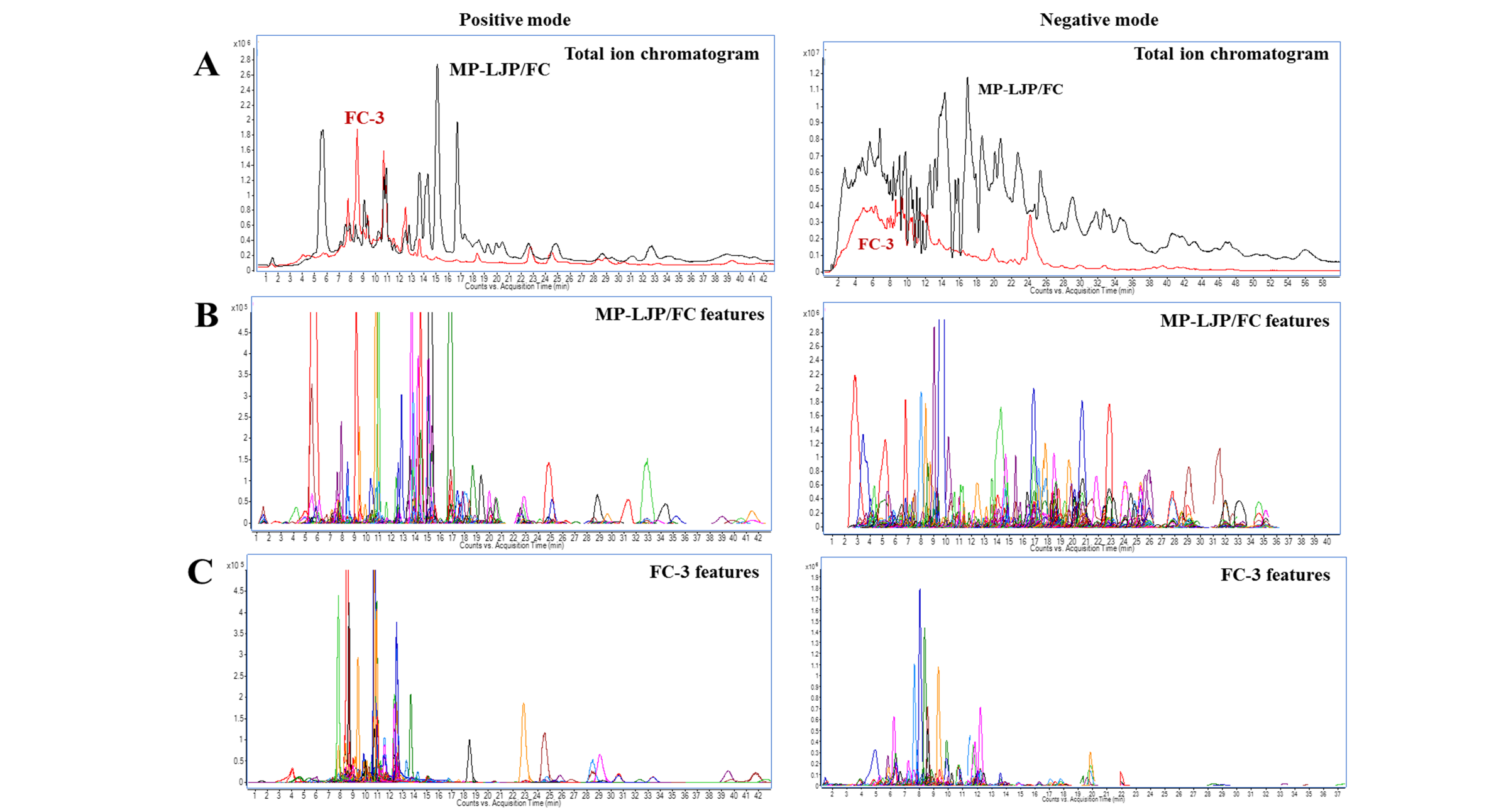


**Supplementary Figure S1: Total ion chromatogram (A) and extracted chromatogram of MP-LJP/FC (B) and FC-3 (C), in positive (left) and negative (right) ionisation modes.**


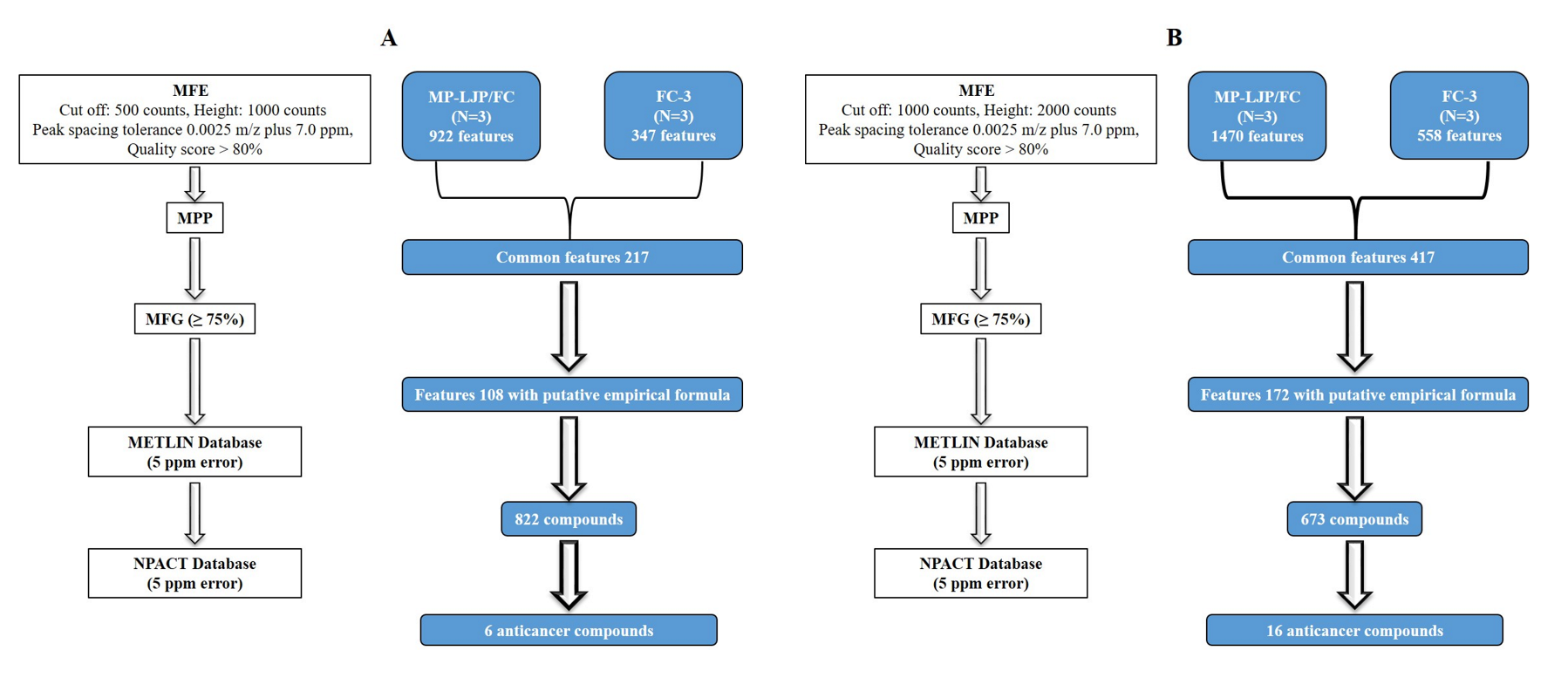


**Supplementary Figure S2:** Schematic workflow for putative identification of FC-3 associated compounds in A) positive and B) negative ion mode.

**Supplementary Table S1. List of 108 discriminatory metabolites in FC-3 (positive ion mode)**

| **S. No.** | **Experimental mass** | **[M+H]^+^** | **Retention time (min.)** | **Empirical formula** | **Error (ppm)^@^** | **Number of hits^#^** | **Putative compounds^$^** |
| --- | --- | --- | --- | --- | --- | --- | --- |
| 1 | 112.1261 | 113.1334 | 8.622 | ND | - | - | - |
| 2 | 166.0275 | 149.0242 | 8.624 | ND | - | - | - |
| 3 | 191.1534 | 192.1608 | 1.52 | ND | - | - | - |
| 4 | 227.2249 | 228.2324 | 6.797 | C_14_ H_29_NO | 0 | 1 | - |
| 5 | 238.2289 | 239.2366 | 7.764 | C_16_H_30_O_2_ | 1 | 34 | Hexadecenoic acid |
| 6 | 255.2563 | 256.264 | 7.577 | C_16_H_33_NO | 1 | 1 | - |
| 7 | 274.1936 | 257.1902 | 3.999 | C_18_H_26_O_2_ | 0 | 2 | - |
| 8 | 284.2718 | 267.2685 | 8.495 | C_18_H_36_O_2_ | 0 | 19 | Stearic acid |
| 9 | 269.2716 | 270.2794 | 7.823 | C_17_H_35_NO | 0 | 1 | - |
| 10 | 278.1534 | 279.1606 | 8.623 | ND | - | - | - |
| 11 | 278.225 | 279.2321 | 4.542 | C_18_H_30_O_2_ | 0 | 78 | Alpha and gamma linolenic acid |
| 12 | 279.2555 | 280.263 | 5.881 | C_18_H_33_NO | 1 | 3 | - |
| 13 | 281.2725 | 282.2799 | 7.759 | C_18_H_35_NO | 2 | 3 | - |
| 14 | 283.2878 | 284.2954 | 8.302 | C_18_H_37_NO | 2 | 1 | - |
| 15 | 284.2356 | 285.2429 | 7.043 | C_17_H_32_O_3_ | 1 | 10 | 1,2,4-trihydroxyheptadec-16-yne |
| 16 | 309.3033 | 310.3109 | 8.458 | C_20_H_39_NO | 1 | 2 | - |
| 17 | 312.2663 | 313.2742 | 12.322 | C_19_H_36_O_3_ | 1 | 5 | - |
| 18 | 314.2455 | 315.2526 | 10.533 | C_18_H_34_O_4_ | 1 | 43 | - |
| 19 | 344.2945 | 327.2912 | 8.089 | ND | - | - | - |
| 20 | 333.2704 | 334.2783 | 7.575 | C_18_H_39_NO_2_S | 1 | 2 | - |
| 21 | 334.2511 | 335.2584 | 10.708 | C_21_H_34_O_3_ | 1 | 21 | - |
| 22 | 335.3186 | 336.3262 | 7.143 | C_22_H_41_NO | 0 | 1 | - |
| 23 | 337.3357 | 338.3431 | 9.366 | C_22_H_43_NO | 4 | 3 | - |
| 24 | 338.2822 | 339.2895 | 7.907 | C_21_H_38_O_3_ | 0 | 1 | - |
| 25 | 340.2427 | 341.2491 | 6.781 | C_20_H_36_O_2_S | 4 | 6 | - |
| 26 | 340.2979 | 341.3052 | 29.27 | C_21_H_40_O_3_ | 0 | 6 | - |
| 27 | 345.3034 | 346.3109 | 8.303 | C_23_H_39_NO | 1 | 5 | - |
| 28 | 359.2871 | 360.2939 | 7.759 | ND | - | - | - |
| 29 | 360.3026 | 361.3099 | 10.207 | C_24_H_40_O_2_ | 0 | 10 | - |
| 30 | 365.3656 | 366.3734 | 10.517 | ND | - | - | - |
| 31 | 368.2939 | 369.3009 | 8.725 | C_23_H_36_N_4_ | 2 | 1 | - |
| 32 | 386.3399 | 369.3364 | 9.441 | C_23_H_46_O_4_ | 0 | 3 | - |
| 34 | 371.319 | 372.3266 | 8.494 | ND | - | - | - |
| 35 | 373.3342 | 374.3421 | 9.209 | C_25_H_43_NO | 0 | 6 | - |
| **S. No.** | **Experimental mass** | **[M+H]^+^** | **Retention time (min.)** | **Empirical formula** | **Error (ppm)^@^** | **Number of hits^#^** | **Putative compounds^$^** |
| 36 | 375.3354 | 376.3429 | 8.494 | ND | - | - | - |
| 37 | 380.3445 | 381.3518 | 12.272 | ND | - | - | - |
| 38 | 382.31 | 383.3171 | 8.908 | C_24_H_38_N_4_ | 3 | 6 | - |
| 39 | 382.3604 | 383.3677 | 10.42 | ND | - | - | - |
| 40 | 384.3394 | 385.3466 | 25.203 | C_27_H_44_O | 0 | 43 | Vitamin D |
| 41 | 388.2624 | 389.2695 | 7.156 | C_25_H_32_N_4_ | 2 | 67 | - |
| 42 | 390.2792 | 391.2865 | 8.623 | ND | - | - | - |
| 43 | 394.3592 | 395.3674 | 10.004 | C_29_H_46_ | 0 | 2 | - |
| 44 | 396.3759 | 397.3833 | 15.013 | C_29_H_48_ | 1 | 1 | - |
| 45 | 400.2834 | 401.2906 | 11.83 | ND | - | - | - |
| 46 | 400.3341 | 401.3415 | 8.003 | C_20_H_44_N_6_S | 0 | 53 | - |
| 47 | 406.3597 | 407.3671 | 24.961 | C_30_H_46_ | 0 | 1 | - |
| 48 | 414.3496 | 415.357 | 13.091 | C_28_H_46_O_2_ | 0 | 21 | - |
| 49 | 415.3491 | 416.3563 | 9.364 | ND | - | - | - |
| 50 | 416.3653 | 417.3726 | 11.891 | C_28_H_48_O_2_ | 0 | 16 | Gamma-Tocopherol |
| 51 | 422.2682 | 423.2753 | 10.387 | C_25_H_34_N_4_O_2_ | 2 | 12 | - |
| 52 | 422.3555 | 423.3627 | 8.951 | C_30_H_46_O | 1 | 1 | - |
| 53 | 424.3692 | 425.3766 | 8.483 | C_30_H_48_O | 2 | 16 | - |
| 54 | 442.4027 | 425.3995 | 11.778 | ND | - | - | - |
| 55 | 442.4028 | 425.3996 | 12.188 | ND | - | - | - |
| 56 | 426.3715 | 427.3788 | 10.535 | C_26_H_50_O_4_ | 1 | 1 | - |
| 57 | 428.3655 | 429.3726 | 14.314 | C_29_H_48_O_2_ | 0 | 23 | - |
| 58 | 428.3661 | 429.3734 | 10.363 | C_29_H_48_O_2_ | 1 | 23 | - |
| 59 | 434.3405 | 435.3477 | 9.381 | C_28_H_42_N_4_ | 1 | 55 | - |
| 60 | 438.3851 | 439.3937 | 11.324 | C_31_H_50_O | 0 | 3 | - |
| 61 | 440.3655 | 441.3728 | 8.81 | C_30_H_48_O_2_ | 0 | 16 | Friedelan-1,3-dione |
| 62 | 444.3608 | 445.3681 | 14.218 | C_29_H_48_O_3_ | 1 | 11 | - |
| 63 | 446.3702 | 447.3779 | 9.946 | ND | - | - | - |
| 64 | 450.3504 | 451.3577 | 11.198 | C_31_H_46_O_2_ | 1 | 1 | - |
| 65 | 454.3813 | 455.3888 | 10.272 | C_31_H_50_O_2_ | 0 | 2 | - |
| 66 | 462.3699 | 463.3774 | 8.156 | C_29_H_50_O_4_ | 1 | 3 | - |
| 67 | 476.3864 | 477.3934 | 13.296 | C_30_H_52_O_4_ | 0 | 2 | - |
| 68 | 478.3766 | 479.3838 | 5.798 | C_28_H_50_N_2_O_4_ | 1 | 1 | - |
| 69 | 488.3503 | 489.3574 | 7.17 | C_30_H_48_O_5_ | 0 | 19 | - |
| 70 | 494.4335 | 495.4405 | 13.058 | ND | - | - | - |
| 71 | 500.3512 | 501.3578 | 7.288 | ND | - | - | - |
| 72 | 522.4648 | 523.4721 | 15.311 | ND | - | - | - |
| 73 | 526.4596 | 527.4668 | 9.963 | C_32_H_62_O_5_ | 0 | 4 | - |
| 74 | 528.383 | 529.3897 | 8.234 | C_34_H_48_N_4_O | 1 | 2 | - |
| 75 | 528.4544 | 529.4616 | 14.142 | C_35_H_60_O_3_ | 0 | 1 | - |
| **S. No.** | **Experimental mass** | **[M+H]^+^** | **Retention time (min.)** | **Empirical formula** | **Error (ppm)^@^** | **Number of hits^#^** | **Putative compounds^$^** |
| 76 | 551.4909 | 534.4882 | 11.313 | C_34_H_65_NO_4_ | 0 | 1 | - |
| 77 | 558.3923 | 541.3892 | 7.683 | ND | - | - | - |
| 78 | 549.3404 | 550.3472 | 10.276 | ND | - | - | - |
| 79 | 550.4969 | 551.5043 | 18.44 | C_35_H_66_O_4_ | 1 | 5 | - |
| 80 | 554.4334 | 555.4406 | 9.093 | C_29_H_58_N_6_O_2_S | - | - | - |
| 81 | 568.4149 | 569.4207 | 8.305 | ND | - | - | - |
| 82 | 574.497 | 575.5033 | 16.68 | C_30_H_66_N_6_O_2_S | 0 | 1 | - |
| 83 | 578.5286 | 579.5359 | 22.845 | ND | - | - | - |
| 84 | 580.4162 | 581.4228 | 8.325 | ND | - | - | - |
| 85 | 588.4733 | 589.4821 | 9.124 | C_41_H_64_S | 0 | 17 | - |
| 86 | 590.4904 | 591.498 | 10.676 | C_37_H_66_O_5_ | 0 | 14 | - |
| 87 | 592.2689 | 593.2762 | 8.992 | C_35_H_36_N_4_O_5_ | 0 | 1 | Pheophorbide a |
| 88 | 594.465 | 595.4724 | 10.711 | ND | - | - | - |
| 89 | 596.48 | 597.4872 | 11.524 | ND | - | - | - |
| 90 | 598.461 | 599.4679 | 10.208 | C_39_H_58_N_4_O | 1 | 3 | - |
| 91 | 606.559 | 607.5664 | 29.065 | ND | - | - | - |
| 92 | 610.4601 | 611.4675 | 9.121 | C_39_H_62_O_5_ | 0 | 6 | - |
| 93 | 612.4761 | 613.4831 | 10.704 | C_39_H_64_O_5_ | 0 | 14 | - |
| 94 | 614.4906 | 615.4978 | 10.121 | C_39_H_66_O_5_ | 0 | 20 | - |
| 95 | 616.5085 | 617.5144 | 12.579 | C_40_H_72_S_2_ | 0 | 22 | - |
| 96 | 624.4408 | 625.4465 | 7.549 | ND | - | - | - |
| 97 | 626.4555 | 627.4624 | 8.268 | ND | - | - | - |
| 98 | 629.5024 | 630.5097 | 12.512 | ND | - | - | - |
| 99 | 650.406 | 633.4013 | 8.211 | C_33_H_62_O_10_S | 2 | 3 | - |
| 100 | 636.2997 | 637.3067 | 7.163 | ND | - | - | - |
| 101 | 640.506 | 641.5137 | 11.976 | C_41_H_68_O_5_ | 0 | 24 | - |
| 102 | 662.4476 | 663.4549 | 13.656 | ND | - | - | - |
| 103 | 667.6479 | 668.6553 | 24.736 | C_42_H_85_NO_4_ | 0 | 5 | - |
| 104 | 678.4237 | 679.4309 | 17.547 | C_44_H_58_N_2_O_2_S | 3 | 6 | - |
| 105 | 709.658 | 710.6656 | 25.373 | ND | - | - | - |
| 106 | 730.4665 | 713.4631 | 8.305 | ND | - | - | - |
| 107 | 734.5338 | 735.541 | 11.83 | ND | - | - | - |
| 108 | 740.4606 | 741.4678 | 13.655 | C_47_H_60_N_6_S | 3 | 6 | - |

ND: Not detected

^@^Error (ppm): the difference between experimental mass and theoretical mass of compound/theoretical mass of compound

^#^Hits obtained from METLIN database

**^$^**Putative compounds from NPACT database

**Supplementary Table S2. List of 172 discriminatory metabolites in FC-3 (negative ion mode)**

| **S. No.** | **Experimental mass** | **[M-H]^-^** | **Retention time (min.)** | **Empirical formula** | **Error (ppm)^@^** | **Number of hits^#^** | **Putative**  **compounds^$^** |
| --- | --- | --- | --- | --- | --- | --- | --- |
| 1 | 208.146 | 189.127 | 2.227 | ND | - | - | - |
| 2 | 208.147 | 207.139 | 5.218 | C_13_H_20_O_2_ | 1 | 14 | - |
| 3 | 210.126 | 209.119 | 5.554 | C_12_H_18_O_3_ | 1 | 14 | - |
| 4 | 220.191 | 219.184 | 6.93 | ND | - | - | - |
| 5 | 228.219 | 227.212 | 7.604 | ND | - | - | - |
| 6 | 232.183 | 231.176 | 5.12 | C_16_H_24_O | 1 | 1 | - |
| 7 | 234.162 | 233.155 | 2.71 | C_15_H_22_O_2_ | 0 | 58 | Isodihydrocostunolide, Buddledin B, 12-Hydroxychiloscyphone |
| 8 | 236.178 | 235.17 | 4.59 | C_15_H_24_O_2_ | 0 | 44 | - |
| 9 | 242.236 | 241.229 | 7.936 | ND | - | - | - |
| 10 | 250.157 | 249.15 | 2.598 | C_15_H_22_O_3_ | 0 | 30 | Viscic acid |
| 11 | 272.247 | 253.229 | 7.822 | ND | - | - | - |
| 12 | 254.237 | 253.23 | 8.059 | ND | - | - | - |
| 13 | 256.253 | 255.245 | 8.347 | ND | - | - | - |
| 14 | 266.168 | 265.161 | 7.913 | C_11_H_26_N_2_O_3_S | 3 | 1 | - |
| 15 | 268.253 | 267.246 | 8.193 | ND | - | - | - |
| 16 | 272.178 | 271.171 | 3.371 | C_18_H_24_O_2_ | 0 | 20 | 3-beta ,12-dihydroxy-13-methyl-6,8,11,13-podocarpatetraen; Octadeca-9,11,13-triynoic acid |
| 17 | 274.194 | 273.187 | 3.821 | C_18_H_26_O_2_ | 3 | 24 | - |
| 18 | 278.154 | 277.147 | 5.657 | ND | - | - | - |
| 19 | 278.189 | 277.182 | 5.12 | C_17_H_26_O_3_ | 2 | 7 | - |
| 20 | 278.237 | 277.229 | 7.615 | ND | - | - | - |
| **S. No.** | **Experimental mass** | **[M-H]^-^** | **Retention time (min.)** | **Empirical formula** | **Error (ppm)^@^** | **Number of hits^#^** | **Putative**  **compounds^$^** |
| 21 | 280.253 | 279.246 | 8.008 | ND | - | - | - |
| 22 | 284.285 | 283.278 | 8.543 | ND | - | - | - |
| 23 | 294.184 | 293.177 | 4.031 | C_18_H_22_N_4_ | 3 | 9 | - |
| 24 | 312.239 | 293.221 | 6.209 | ND | - | - | - |
| 25 | 296.243 | 295.236 | 6.268 | ND | - | - | - |
| 26 | 300.277 | 299.269 | 6.97 | ND | - | - | - |
| 27 | 300.281 | 299.274 | 8.541 | ND | - | - | - |
| 28 | 308.277 | 307.27 | 8.855 | ND | - | - | - |
| 29 | 310.216 | 309.208 | 5.188 | C_19_H_26_N_4_ | 3 | 25 | - |
| 30 | 310.217 | 309.209 | 5.728 | ND | - | - | - |
| 31 | 310.223 | 309.216 | 6.24 | ND | - | - | - |
| 32 | 310.288 | 309.281 | 9.573 | C_20_H_38_O_2_ | 3 | 42 | - |
| 33 | 312.114 | 311.107 | 3.476 | C_22_H_16_O_2_ | 0 | 5 | - |
| 34 | 312.191 | 311.184 | 8.179 | ND | - | - | - |
| 35 | 312.195 | 311.188 | 3.802 | C_18_H_24_N_4_O | 0 | 1 | - |
| 36 | 312.231 | 311.223 | 5.251 | C_18_H_32_O_4_ | 1 | 51 | - |
| 37 | 314.247 | 313.24 | 6.623 | ND | - | - | - |
| 38 | 314.258 | 313.25 | 7.05 | ND | - | - | - |
| 39 | 316.205 | 315.198 | 5.339 | C_20_H_28_O_3_ | 2 | 57 | 8,14-epoxide; Caracasine acid; Multidione; Rabdoumbrosanin; (+)-7-Oxo-13-epi-pimara-14,15-dien-18-oic acid; (+)-7-Oxo-13-epi-pimara-8,15-dien-18-oic acid; Taiwaniaqu--inone G |
| 40 | 316.261 | 315.253 | 5.981 | C_18_H_36_O_4_ | 2 | 18 | - |
| 41 | 324.194 | 323.187 | 3.001 | C_18_H_28_O_5_ | 0 | 5 | 3-alpha-acetoxydiversifolol |
| 42 | 324.231 | 323.224 | 5.797 | C_20_H_28_N_4_ | 2 | 5 | - |
| 43 | 326.207 | 325.2 | 8.604 | ND | - | - | - |
| 44 | 326.211 | 325.203 | 4.825 | C_19_H_26_N_4_O | 3 | 8 | - |
| **S. No.** | **Experimental mass** | **[M-H]^-^** | **Retention time (min.)** | **Empirical formula** | **Error (ppm)^@^** | **Number of hits^#^** | **Putative**  **compounds^$^** |
| 45 | 327.234 | 326.227 | 5.962 | ND | - | - | - |
| 46 | 328.313 | 327.306 | 8.249 | ND | - | - | - |
| 47 | 328.316 | 327.309 | 11.56 | ND | - | - | - |
| 48 | 330.239 | 329.232 | 3.292 | C_14_H_30_N_6_O_3_ | 4 | 15 | - |
| 49 | 335.256 | 334.249 | 7.097 | ND | - | - | - |
| 50 | 336.193 | 335.186 | 2.557 | C_19_H_28_O_5_ | 0 | 1 | - |
| 51 | 340.214 | 339.207 | 9.019 | C_13_H_32_N_4_O_4_S | 2 | 1 | - |
| 52 | 340.334 | 339.327 | 12.01 | C_22_H_44_O_2_ | 1 | 18 | - |
| 53 | 342.221 | 341.214 | 5.442 | C_22_H_30_O_3_ | 4 | 10 | - |
| 54 | 354.349 | 353.342 | 13 | C_23_H_46_O_2_ | 1 | 14 | - |
| 55 | 356.33 | 355.323 | 11.56 | C_22_H_44_O_3_ | 3 | 5 | - |
| 56 | 363.292 | 362.284 | 7.812 | ND | - | - | - |
| 57 | 363.948 | 362.941 | 1.512 | ND | - | - | - |
| 58 | 367.277 | 366.271 | 7.096 | ND | - | - | - |
| 59 | 368.365 | 367.358 | 14.12 | C_24_H_48_O_2_ | 0 | 19 | - |
| 60 | 374.181 | 373.173 | 5.101 | ND | - | - | - |
| 61 | 376.195 | 375.187 | 5.544 | ND | - | - | - |
| 62 | 376.298 | 375.291 | 7.806 | C_17_H_40_N_6_OS | 2 | 44 | - |
| 63 | 377.308 | 376.301 | 8.157 | ND | - | - | - |
| 64 | 386.117 | 385.11 | 2.593 | C_25_H_14_N_4_O | 3 | 2 | - |
| 65 | 390.316 | 389.308 | 7.815 | C_22_H_46_O_3_S | 4 | 5 | - |
| 66 | 391.325 | 390.318 | 8.542 | ND | - | - | - |
| 67 | 392.239 | 391.231 | 9.609 | C_16_H_28_N_10_O_2_ | 3 | 2 | - |
| 68 | 393.294 | 392.287 | 7.036 | ND | - | - | - |
| 69 | 396.326 | 395.319 | 9.659 | ND | - | - | - |
| 70 | 396.396 | 395.389 | 17 | C_26_H_52_O_2_ | 0 | 16 | - |
| 71 | 400.313 | 399.306 | 7.507 | C_30_H_40_ | 0 | 1 | - |
| 72 | 404.313 | 403.306 | 9.229 | ND | - | - | - |
| 73 | 404.333 | 403.326 | 8.54 | ND | - | - | - |
| 74 | 405.112 | 404.105 | 1.381 | ND | - | - | - |
| 75 | 424.302 | 405.284 | 8.716 | ND | - | - | - |
| 76 | 410.263 | 409.256 | 9.295 | ND | - | - | - |
| 77 | 410.376 | 409.368 | 11.85 | C_26_H_50_O_3_ | 0 | 1 | - |
| 78 | 412.283 | 411.275 | 5.987 | C_23_H_40_O_6_ | 0 | 1 | - |
| 79 | 412.355 | 411.348 | 11.98 | C_25_H_48_O_4_ | 0 | 2 | - |
| 80 | 412.357 | 411.35 | 11.57 | C_26_H_44_N_4_ | 4 | 2 | - |
| 81 | 412.389 | 411.382 | 10.14 | ND | - | - | - |
| 82 | 414.332 | 413.325 | 7.846 | ND | - | - | - |
| 83 | 418.35 | 417.342 | 8.548 | ND | - | - | - |
| 84 | 422.115 | 421.108 | 1.701 | C_27_H_18_O_5_ | 0 | 2 | - |
| 85 | 422.255 | 421.247 | 8.257 | ND | - | - | - |
| **S. No.** | **Experimental mass** | **[M-H]^-^** | **Retention time (min.)** | **Empirical formula** | **Error (ppm)^@^** | **Number of hits^#^** | **Putative**  **compounds^$^** |
| 86 | 427.978 | 426.971 | 5.735 | ND | - | - | - |
| 87 | 430.294 | 429.287 | 5.269 | ND | - | - | - |
| 88 | 433.37 | 432.363 | 10.08 | C_23_H_51_N_3_O_2_S | 4 | 2 | - |
| 89 | 434.253 | 433.246 | 8.969 | ND | - | - | - |
| 90 | 438.104 | 437.097 | 1.362 | ND | - | - | - |
| 91 | 450.159 | 449.151 | 5.303 | ND | - | - | - |
| 92 | 450.161 | 449.153 | 5.801 | ND | - | - | - |
| 93 | 450.168 | 449.161 | 6.232 | C_26_H_26_O_7_ | 0 | 9 | - |
| 94 | 452.371 | 451.364 | 8.524 | ND | - | - | - |
| 95 | 456.11 | 455.102 | 1.488 | ND | - | - | - |
| 96 | 458.38 | 457.373 | 8.993 | ND | - | - | - |
| 97 | 463.389 | 462.383 | 9.398 | ND | - | - | - |
| 98 | 474.291 | 473.283 | 13.52 | ND | - | - | - |
| 99 | 482.274 | 481.267 | 8.842 | ND | - | - | - |
| 100 | 486.253 | 485.245 | 7.989 | ND | - | - | - |
| 101 | 490.267 | 489.26 | 17.3 | ND | - | - | - |
| 102 | 491.359 | 490.353 | 12.63 | ND | - | - | - |
| 103 | 499.923 | 498.915 | 1.504 | ND | - | - | - |
| 104 | 504.317 | 503.31 | 2.831 | C_32_H_44_N_2_OS | 0 | 1 | - |
| 105 | 508.297 | 507.289 | 8.778 | ND | - | - | - |
| 106 | 509.371 | 508.364 | 12.9 | C_26_H_43_N_11_ | 3 | 1 | - |
| 107 | 510.303 | 509.295 | 9.514 | ND | - | - | - |
| 108 | 524.289 | 523.28 | 8.937 | C_23_H_48_N_4_O_3_S_3_ | 2 | 1 | - |
| 109 | 526.465 | 525.458 | 10.06 | ND | - | - | - |
| 110 | 546.328 | 527.309 | 7.206 | ND | - | - | - |
| 111 | 530.333 | 529.325 | 7.237 | ND | - | - | - |
| 112 | 546.464 | 545.457 | 13.9 | C_35_H_62_O_4_ | 0 | 2 | - |
| 113 | 556.299 | 555.292 | 8.909 | C_27_H_44_N_2_O_10_ | 4 | 1 | - |
| 114 | 556.449 | 555.442 | 12.14 | ND | - | - | - |
| 115 | 568.564 | 567.556 | 9.279 | ND | - | - | - |
| 116 | 572.316 | 571.308 | 9.124 | ND | - | - | - |
| 117 | 578.304 | 577.297 | 8.178 | ND | - | - | - |
| 118 | 591.377 | 590.369 | 12.44 | ND | - | - | - |
| 119 | 593.297 | 592.289 | 9.17 | ND | - | - | - |
| 120 | 596.325 | 595.318 | 8.659 | ND | - | - | - |
| 121 | 600.474 | 599.467 | 12.58 | C_34_H_68_N_2_O_2_S_2_ | 1 | 7 | - |
| 122 | 601.504 | 600.496 | 18.04 | ND | - | - | - |
| 123 | 610.542 | 609.535 | 7.935 | ND | - | - | - |
| 124 | 614.577 | 613.57 | 8.496 | ND | - | - | - |
| 125 | 618.442 | 617.435 | 8.188 | ND | - | - | - |
| 126 | 624.29 | 623.282 | 8.365 | ND | - | - | - |
| **S. No.** | **Experimental mass** | **[M-H]^-^** | **Retention time (min.)** | **Empirical formula** | **Error (ppm)^@^** | **Number of hits^#^** | **Putative**  **compounds^$^** |
| 127 | 624.47 | 623.462 | 8.827 | ND | - | - | - |
| 128 | 629.535 | 628.528 | 22.3 | ND | - | - | - |
| 129 | 635.897 | 634.89 | 1.506 | ND | - | - | - |
| 130 | 638.398 | 637.388 | 9.637 | C_43_H_58_S_2_ | 0 | 4 | - |
| 131 | 639.479 | 638.474 | 10.18 | ND | - | - | - |
| 132 | 644.253 | 643.246 | 11.35 | ND | - | - | - |
| 133 | 645.467 | 644.464 | 12.36 | C_44_H_59_N_3_O | 3 | 2 | - |
| 134 | 652.443 | 651.437 | 7.274 | C_35_H_64_N_4_O_3_S_2_ | 4 | 1 | - |
| 135 | 653.535 | 652.528 | 19.95 | ND | - | - | - |
| 136 | 653.595 | 652.588 | 16.21 | ND | - | - | - |
| 137 | 654.341 | 653.333 | 8.612 | ND | - | - | - |
| 138 | 655.611 | 654.604 | 17.69 | C_40_H_81_NO_5_ | 1 | 3 | - |
| 139 | 657.565 | 656.557 | 28.28 | ND | - | - | - |
| 140 | 662.447 | 661.44 | 13.52 | ND | - | - | - |
| 141 | 662.454 | 661.447 | 7.754 | ND | - | - | - |
| 142 | 667.61 | 666.603 | 17.81 | ND | - | - | - |
| 143 | 669.586 | 668.579 | 11.91 | ND | - | - | - |
| 144 | 669.59 | 668.583 | 11.59 | ND | - | - | - |
| 145 | 669.626 | 668.619 | 19.53 | C_41_H_83_NO_5_ | 0 | 1 | - |
| 146 | 672.568 | 671.56 | 18.52 | C_47_H_76_S | 2 | 22 | - |
| 147 | 674.223 | 673.216 | 11.71 | C_35_H_38_N_4_O_6_S_2_ | 3 | 1 | - |
| 148 | 639.616 | 674.585 | 19.27 | C_40_H_81_NO_4_ | 0 | 1 | - |
| 149 | 681.627 | 680.621 | 19.72 | ND | - | - | - |
| 150 | 683.642 | 682.635 | 21.78 | C_42_H_85_NO_5_ | 0 | 2 | - |
| 151 | 684.435 | 683.428 | 7.193 | ND | - | - | - |
| 152 | 685.62 | 684.614 | 19.28 | ND | - | - | - |
| 153 | 695.641 | 694.634 | 22 | ND | - | - | - |
| 154 | 697.655 | 696.648 | 24.39 | C_39_H_83_N_7_O_3_ | 4 | 1 | - |
| 155 | 698.449 | 697.442 | 6.969 | ND | - | - | - |
| 156 | 698.453 | 697.444 | 7.349 | ND | - | - | - |
| 157 | 699.599 | 698.594 | 16.21 | ND | - | - | - |
| 158 | 700.408 | 699.401 | 5.725 | ND | - | - | - |
| 159 | 707.639 | 706.633 | 20.07 | ND | - | - | - |
| 160 | 709.654 | 708.647 | 24.69 | ND | - | - | - |
| 161 | 711.636 | 710.629 | 21.73 | ND | - | - | - |
| 162 | 724.632 | 723.624 | 19.71 | ND | - | - | - |
| 163 | 725.649 | 724.642 | 24.38 | ND | - | - | - |
| 164 | 727.452 | 726.444 | 7.351 | ND | - | - | - |
| 165 | 727.632 | 726.626 | 19.72 | ND | - | - | - |
| 166 | 729.646 | 728.64 | 21.78 | ND | - | - | - |
| 167 | 740.4 | 739.392 | 4.418 | ND | - | - | - |
| **S. No.** | **Experimental mass** | **[M-H]^-^** | **Retention time (min.)** | **Empirical formula** | **Error (ppm)^@^** | **Number of hits^#^** | **Putative**  **compounds^$^** |
| 168 | 740.467 | 739.458 | 7.39 | C_41_H_68_N_6_S_3_ | 3 | 6 | - |
| 169 | 740.661 | 739.654 | 24.4 | ND | - | - | - |
| 170 | 741.575 | 740.569 | 11.62 | C_42_H_79_NO_9_ | 0 | 5 | - |
| 171 | 744.619 | 743.613 | 19.74 | ND | - | - | - |
| 172 | 745.459 | 744.452 | 6.988 | ND | - | - | - |

ND: Not detected

^@^Error (ppm): the difference between experimental mass and theoretical mass of compound/theoretical mass of compound

^$^Hits obtained from METLIN database

**^$^**Putative compounds from NPACT database
